# Burden of rheumatoid arthritis in India from 1990 to 2021: insights from the Global Burden of Disease Database

**DOI:** 10.3389/fmed.2025.1526218

**Published:** 2025-02-14

**Authors:** Wei Shi, Xinyu Liang, Huafeng Zhang, Hui Li

**Affiliations:** Department of Orthopedics, Tianjin Medical University General Hospital, Tianjin, China

**Keywords:** epidemiology, prevalence, incidence, disease burden, rheumatoid arthritis

## Abstract

**Objective:**

Rheumatoid arthritis (RA) significantly impacts individual health and society. This study aims to analyze the burden of RA in India from 1990 to 2021 and provide predictions to inform national prevention and control strategies.

**Methods:**

Utilizing data from the 2021 Global Burden of Disease (GBD) database, this study describes changes in incidence, prevalence, and disability-adjusted life years (DALY) related to RA in India from 1990 to 2021, while also observing variations across 31 geographical regions.

**Results:**

From 1990 to 2021 in India, the incidence, prevalence, and DALY rates of RA showed an increasing trend, with all age-standardized rates being significantly higher in females than in males. The age group of 65–69 years showed the highest incidence rate, while the prevalence peaked at 75–79 years. The forecast results indicate that the age-standardized incidence and DALY rates of RA in India will show an upward trend from 2022 to 2036. There were considerable differences in prevalence across different regions. In 2021, the highest male prevalence was in Uttarakhand, while Goa had the lowest. For females, Tamil Nadu had the highest prevalence, and Madhya Pradesh had the lowest. Overall, areas with high Socio-Demographic Index (SDI) showed a higher disease burden, whereas Kerala, despite a higher SDI, had the lowest burden.

**Conclusion:**

Using the GBD database, our findings show that the disease burden of RA in India is on the rise from 1990 to 2021. The prevalence of RA in different regions of India is significantly different, which may be related to local economy and development. The high prevalence of RA in Indian women requires more attention to the early diagnosis and treatment of RA.

## Introduction

Rheumatoid arthritis (RA) is an idiopathic disease characterized by inflammation in synovial tissues of joints, cartilage, and bones (1, 2). RA is thought to be driven by genetic and epigenetic factors, but environmental factors such as cigarette smoke and dust exposure also play crucial roles (3–5). Uncontrolled RA can lead to joint degradation, severe disability, reduced quality of life, comorbidities, and premature death, posing a significant societal burden (6–8). Approximately 70% of RA patients in India suffer from anxiety and depression (9). A cross-sectional, hospital-based study conducted in a tertiary care private multi-specialty hospital in Tamil Nadu revealed that the average annual medical expenditure for treating RA was 44,700 rupees ($543), with a median of 39,210 rupees ($476) (10). The global prevalence of RA varies, with an estimated 0.5 to 1% of adults affected (1, 11). The prevalence is generally higher in industrialized countries, possibly due to environmental risk factors, genetic predispositions, and underreporting in other regions (12). Recent reviews indicate that the prevalence of RA in India ranges from 0.28 to 0.7%, based on only four studies (13). There is a lack of nationwide epidemiological research on RA in India.

The Global Burden of Disease (GBD) database, established by the Global Burden of Disease Study, is a comprehensive repository maintained by the Institute for Health Metrics and Evaluation (IHME). It systematically quantifies and assesses the impact of major diseases, injuries, and health risk factors globally, regionally, and nationally (14, 15). This study leverages GBD 2021 data to analyze the incidence and prevalence of RA in India and estimates the disease burden and trends across different age groups, providing a basis for developing effective RA prevention and control strategies.

## Materials and methods

### Data source

This study uses data from GBD 2019, analyzing data from 1990 to 2021 for both India and globally. The GBD database, maintained by the IHME, assesses the burden of 389 diseases or injuries and 88 risk factors across 204 countries. The GBD database is classified and defined based on the international classification of diseases (ICD) system. The codes for RA are ICD-9-714 and ICD-10-M05, M06, M08 (16, 17).

### Search strategy

Data were retrieved using the GBD Result Tool available on the IHME website.^1^ The search was conducted under the “GBD Estimate” tab by selecting “Rheumatoid arthritis,” region, age, time, and study indicators. India comprises 31 geographical units: 28 states, the union territory of Delhi, Jammu and Kashmir combined with Ladakh, and other small union territories combined (Chandigarh, Dadra and Nagar Haveli, Daman and Diu, Puducherry, Lakshadweep, and the Andaman and Nicobar Islands).

### Basic indicators

The report provides data on the incidence, prevalence, age-standardized incidence rates (per 100,000 persons), and age-standardized prevalence rates (per 100,000 persons) of RA in India from 1990 to 2021, broken down by year, gender, and age group. It also uses age-standardized disability-adjusted life years (DALY) rates (per 100,000 persons) to measure the disease burden caused by RA. The meaning of DALY is the loss of healthy life years due to early death and disability caused by diseases. The DALY consists mainly of years lived with disability (YLDs) and years of life lost (YLLs). The Autoregressive Integrated Moving Average model (ARIMA) was employed to predict RA incidence and DALY from 2022 to 2036 in India. Socio-Demographic Index (SDI) is a comprehensive indicator used to measure the development status of a country or region and is strongly correlated with health outcomes. The study also reports the prevalence rates across 31 geographical units and analyzes the relationship between the SDI and DALY rates in India.

### Statistical analysis

Our data comes from the GBD database, which provides data on the prevalence of diseases in different years, as well as disability-adjusted life years. We carefully reviewed the data to illustrate the incidence, prevalence and DALY of RA across different age groups and genders in India. Data were age standardized for analysis of morbidity, prevalence, and DLAYs to eliminate age composition disruptions. We use R 4.3.3 software to analyze and visualize the downloaded data.

As all GBD studies are conducted using publicly available data, no ethical approval was required.

## Results

### Prevalence characteristics of RA in India

**FIGURE 1:**
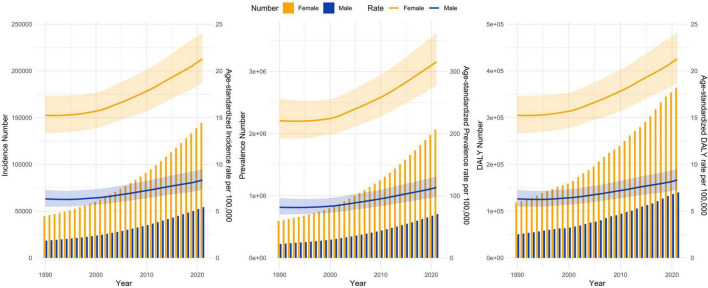
Incidence, prevalence, DALY, and their age-standardized rates (per 100,000 persons) of rheumatoid arthritis by gender from 1990 to 2021. DALY, disability-adjusted life years.

From 1990 to 2021, the incidence, prevalence, and DALY rates of RA in India have shown an increasing trend (Figure 1). All age-standardized rates were significantly higher in females than in males. In 2021, the incidence, prevalence, and DALY rates were 2.76–3.38 times higher than in 1990, with female incidence, prevalence, and DALY rates being 2.60–2.93 times those of males. The number of males affected by RA across all age groups increased from 224,505 in 1990 to 704,401 in 2021, while the number of females increased from 594,068 to 2,062,348. The age-standardized incidence rate (per 100,000 persons) in males increased from 6.32 in 1990 to 8.33 in 2021, while in females, it increased from 15.25 to 21.28. The age-standardized prevalence rate (per 100,000 persons) in males increased from 81.47 in 1990 to 113.32 in 2021, while in females, it increased from 220.81 to 315.44. The age-standardized DALY rate (per 100,000 persons) in males increased from 21.01 in 1990 to 23.94 in 2021, while in females, it increased from 49.19 to 57.07 (Table 1).

**TABLE 1:** Incidence, prevalence, and DALY of rheumatoid arthritis in India, 1990 and 2021.

		Number (95% uncertainty interval)			Age standardized rate (95% uncertainty interval)			
		**Both**	**Male**	**Female**	**Both**	**Male**	**Female**	**Ratio (F/M)**
Incidence	1990	63,047 (54,893, 72,108)	18,471 (15,934, 21,459)	44,576 (39,077, 50,936)	10.61 (9.28, 12.1)	6.32 (5.49, 7.29)	15.25 (13.35, 17.36)	2.41
	2021	198,511 (174,796, 226,624)	54,281 (47,401, 62,166)	144,230 (126,982, 163,796)	14.82 (13.07, 16.89)	8.33 (7.3, 9.56)	21.28 (18.72, 24.07)	2.66
	Ratio (2021/1990)	3.15	2.94	3.24	1.40	1.32	1.40	
Prevalence	1990	818,573 (697,518, 969,037)	224,505 (189,069, 270,853)	594,068 (504,184, 698,431)	148.83 (129.7, 173.4)	81.47 (69.97, 96.34)	220.81 (191.84, 256.39)	2.65
	2021	2,766,749 (2,412,596, 3,215,435)	704,401 (604,952, 832,537)	2,062,348 (1,806,861, 2,381,639)	216.1 (190.08, 248.13)	113.32 (98.65, 131.21)	315.44 (277.86, 362.08)	2.93
	Ratio (2021/1990)	3.38	3.14	3.47	1.45	1.40	1.43	
DALY	1990	169,250 (128,067, 218,649)	50,602 (34,636, 65,404)	118,649 (89,158, 154,730)	34.73 (26.41, 44.44)	21.01 (14.29, 27.38)	49.19 (37.72, 63.55)	2.34
	2021	503,360 (382,564, 638,990)	139,840 (100,234, 179,825)	363,521 (274,638, 465,938)	40.88 (31.65, 51.51)	23.94 (17.21, 30.3)	57.07 (43.56, 73.44)	2.60
	Ratio (2021/1990)	2.97	2.76	3.06	1.17	1.14	1.16	

DALY, disability-adjusted life years.

**FIGURE 2:**
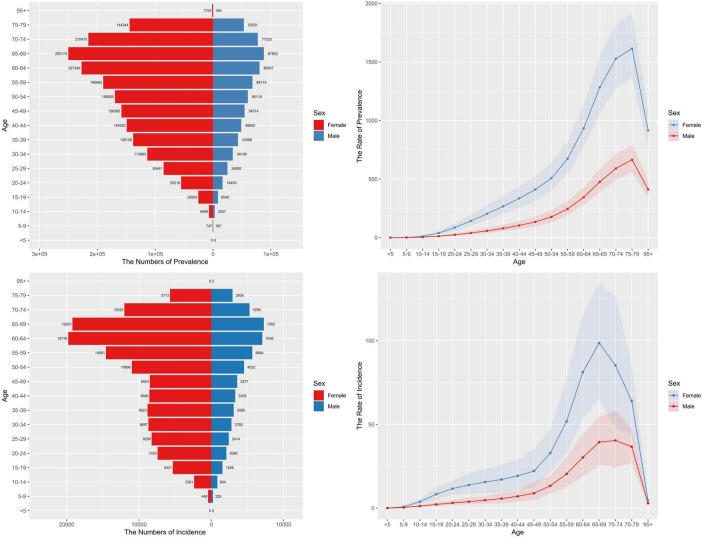
Number and rate (per 100,000 persons) of rheumatoid arthritis cases by age in 2021. DALY, disability-adjusted life years.

**FIGURE 3:**
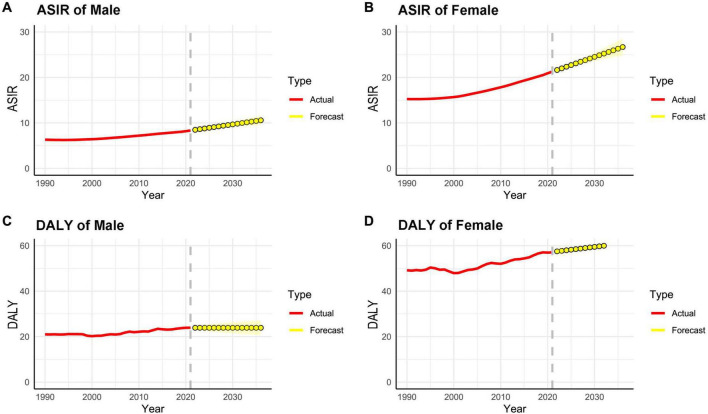
ASIR **(A,B)** and DALY **(C,D)** (per 100,000 persons) of rheumatoid arthritis predicted from 2022 to 2036 using ARIMA forecasting. ASIR, age-standardized incidence rate; DALY, disability-adjusted life years.

The age group of 65–69 years showed the highest incidence rate, while the prevalence peaked at 75–79 years (Figure 2). ARIMA predictions suggest that from 2022 to 2036, incidence and DALY rate, will continue to rise, with a more stable trend in males compared to females (Figure 3).

### Geographical characteristics of RA prevalence in India

**FIGURE 4:**
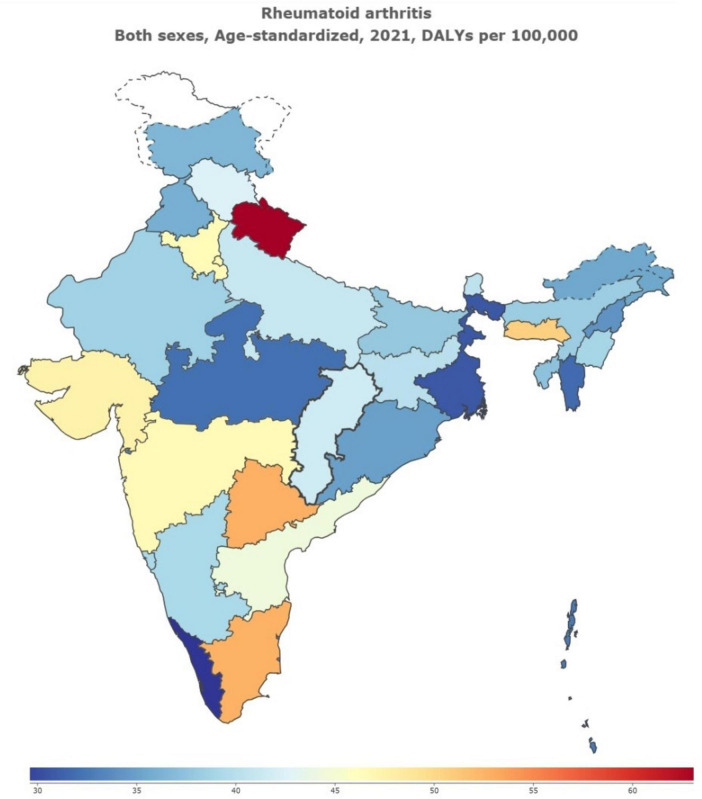
DALYs of Rheumatoid Arthritis in 31 Geographical Regions of India of 2021. DALY, disability-adjusted life years.

**FIGURE 5:**
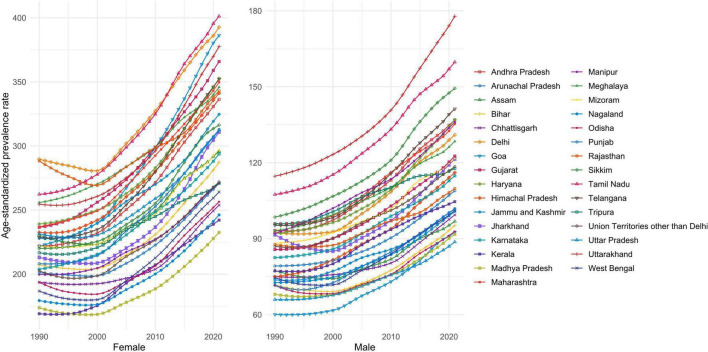
Age-standardized prevalence rates (per 100,000 persons) of rheumatoid arthritis across 31 geographic regions from 1990 to 2021.

**FIGURE 6:**
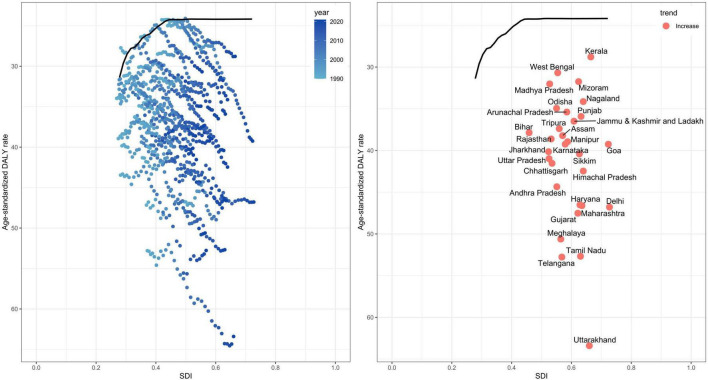
Relationship between age-standardized DALY rates (per 100,000 persons) of rheumatoid arthritis from 1990 to 2021 across 30 geographic regions and the SDI. DALY, disability-adjusted life years; SDI, Socio-Demographic Index.

We observed the DALYs in 31 geographical regions of India (Figure 4). The highest DALYs are in Uttarakhand, followed by Tamil Nadu and Telangana. Significant differences in prevalence were observed across 31 geographical regions (Figure 5). In 2021, Uttarakhand had the highest age-standardized prevalence rate (per 100,000 persons) among males (177.86), while Goa had the lowest (88.74). Among females, Tamil Nadu had the highest rate (401.26), while Madhya Pradesh had the lowest (232.53). Delhi ranked second in female prevalence (392.80) and ninth in male prevalence (131.10). Due to the lack of SDI data for the other small union territories combined, only 30 regions were analyzed (Figure 6). Overall, areas with high SDI showed a higher disease burden, with age-standardized DALY rates (per 100,000 persons) in all 30 regions in 2021 higher than in 1990. The highest DALY rate in 2021 was observed in Uttarakhand (63.40), while the lowest was in Kerala (28.79), despite both regions having similar SDI levels. Interestingly, Delhi, the region with the highest SDI, had a DALY rate (46.79) significantly higher than Goa, the region with the second-highest SDI (39.25).

## Discussion

This study provides a systematic overview of the burden of RA from 1990 to 2021 across India and its states. The age-standardized prevalence, incidence, and DALY rates of RA are rising across India, with a more pronounced increase from 2019 to 2021, and are predicted to continue rising over the next decade. Female rates are consistently higher and increase with age, varying across different states in India. These findings confirm that RA has become a significant public health concern in India.

Female RA incidence rates are 2.6–2.9 times higher than those in males. This gender difference may stem from multiple factors such as physiological differences, sociocultural influences, and lifestyle factors. Females may be more susceptible to autoimmune diseases due to differences in immune system functioning compared to males (18). Estrogen may play a promoting role in RA pathogenesis, while male hormone testosterone may have a protective effect (19–21). In the socio-cultural context of India, women may face greater barriers to accessing healthcare, such as unequal distribution of medical resources and prioritization of male family members’ health, which could lead to delayed diagnosis and treatment for female RA patients. This may result in higher prevalence and disease burden (22, 23). Indian women may also be more affected by certain lifestyle factors, such as a higher burden of household chores, fewer opportunities for physical activity, and potentially poor nutrition associated with poverty and low education levels (24–27).

In the analysis of the global burden of RA, the same trend was shown, namely, the proportion of women with RA was higher (28). Furthermore, in our results, we found that the prevalence of RA in India peaked at 75–79 years of age, again consistent with the results of the global RA burden, showing a clear age-specific pattern. Interestingly, however, our results show that the incidence is highest in the 65–69 age group, which may indicate that there is some delay in the diagnosis of RA in India, or that early diagnosis and treatment of RA need to be strengthened. In addition, the results of the global analysis show that RA is highly correlated with SDI, which also explains the differences in the prevalence of RA between Indian states. However, in some non-high-income regions, the prevalence of RA is significantly lower than in India, and whether this is related to the level of regional diagnosis and regional living standards needs further discussion and research. As for the change in prevalence, the reason for the increase in prevalence in recent years is also the change in diagnostic criteria. In 2010, the American College of Rheumatology (ACR) and the European League against Rheumatism (EULAR) jointly issued a new classification standard for RA. At the heart of the guidelines is a desire to be able to diagnose and intervene earlier in RA, whereas previous criteria were more inclined to diagnose “confirmed RA.” Therefore, the prevalence may increase (29).

The prevalence of RA varies considerably across different geographical unit in India, with regions like Uttarakhand and Tamil Nadu showing significantly higher prevalence rates compared to states like Goa and Madhya Pradesh. These disparities can be partially attributed to differences in the SDI and environmental factors. Environmental factors such as climate, dietary habits, and levels of pollution exposure in different regions may also influence the incidence of RA (30, 31). For instance, the cold and damp climates in certain areas may exacerbate RA symptoms, while regions with higher levels of industrialization might face increased pollution exposure, contributing to higher RA incidence. In regions with higher SDI, RA may be diagnosed more frequently, which does not necessarily indicate a lighter RA burden in low SDI regions. On the contrary, the actual burden in these areas might be underestimated due to inadequate diagnosis and treatment. It is noteworthy that Kerala, despite having a higher SDI, has the lowest disease burden, largely due to its efficient public health system and high-quality medical services. Kerala’s public health system is widely accessible, with well-developed primary healthcare facilities and a relatively equitable distribution of medical resources (32–34). This is a model worth emulating in regions like Uttarakhand. The economic gap leads to disparities in medical-related services among different regions. For instance, the economy can directly affect the development of hospitals and medical technologies in a region, thereby influencing the diagnosis and active treatment of RA. Moreover, the economy also impacts the educational level of a region. Regions with a higher educational level are more helpful in self-protection and early diagnosis of RA compared to those with a lower educational level (35, 36).

Given the increasing burden of RA in India and the pronounced differences across gender and regions, public health strategies should focus on the following areas: (1). Enhancing Identification and Treatment of RA Among Women: The identification and treatment of RA in women should be a priority, especially in rural and low-SDI regions where healthcare resources are limited. This can be achieved through the enhanced training of healthcare professionals and by raising public awareness about RA (37, 38). (2). Regional Health Planning: Due to the significant variation in RA burden across different regions, health policies need to be more flexible and adapted to the specific circumstances of each state. For instance, regions with a high burden, such as Uttarakhand and Delhi, require more intensive prevention and management strategies (13). (3). Promoting Early Diagnosis and Intervention: Early diagnosis and intervention are crucial in reducing the long-term burden of RA. The government should consider expanding RA screening programs, particularly among high-risk groups such as women and the elderly, and improving patients’ self-management capabilities (13, 39). (4). Research and Data Collection: Although substantial progress has been made, further support is necessary for epidemiological research on RA in India. This research is essential to better understand the specific factors contributing to gender and regional disparities. Increased research efforts will aid in developing more targeted interventions.

Our study still has some limitations, for example, data may be scarce for some states, so differences between different Indian states need to be explained more rigorously. In addition, our research data comes from 1990 to 2021, and our calculation and prediction are all based on this period. For future prediction, more recent data or updated data may be required to calculate more accurately.

## Conclusion

This study, based on GBD 2021 data, reveals a growing burden of RA in India from 1990 to 2021, with the trend expected to continue. Notably, the burden is higher among females, and there are considerable regional differences in prevalence. These findings suggest that enhanced early diagnosis and treatment, especially in females and high-burden regions, are critical to reducing the disease’s impact. Efforts should focus on improving healthcare accessibility and providing targeted interventions to reduce the overall burden of RA in India.

## Data Availability

Publicly available datasets were analyzed in this study. This data can be obtained from the IHME website at https://vizhub.healthdata.org/gbd-results/.

## Footnotes

1
https://vizhub.healthdata.org/gbd-results/
